# Die Konturen des Nicht-Wissens im Superwahljahr 2021: Wählen in Zeiten der Pandemie

**DOI:** 10.1007/s41358-021-00253-8

**Published:** 2021-03-16

**Authors:** Karl-Rudolf Korte

**Affiliations:** NRW School of Governance, Universität Duisburg-Essen, Lotharstraße 53, 47057 Duisburg, Deutschland

Wir wissen, dass Attraktivität im Wahlkampf Prozentwerte bringt (Gaßner et al. 2019). Aber wie attraktiv wirkt man auf digitalen Kacheln? Darüber wissen wir viel weniger. In Deutschland zählen bei der Stimmabgabe besonders Sachkompetenz, Glaubwürdigkeit, Führungsqualität sowie – erst an vierter Positionierung – persönliche Sympathien. Auch die Wahlkampfforschung hat Probleme, unter den Bedingungen der pandemisch bedingten Distanz, langgehegte Erkenntnisse einfach fortzuschreiben. Reichen in einer durch die Corona-Politik extrem erschöpften Republik die klassischen Ansätze des Wahlkampf-Dreischritts aus: begrenzte Aggressivität, Sichrheitsbotschaften, Zukunftskompetenz (Korte 2021)?

Wahlen sind ein verläßlicher Gradmesser des Vertrauens.^1^ Welcher Partei, welcher Kandidatin, welchem Kandidaten schenken wir persönliches Zutrauen beim Lösen wichiger Probleme? Das Vertrauens-Reservoir ist im Jahr 2021 herausgefordert. Die Distanz-Demokratie provoziert. Damit ist nicht der Widerstand einer stets kleinen Minderheit gegen die Corona-Maßnahmen gemeint. Vielmehr provoziert uns täglich die überlebensnotwendige Übersetzung demokratischer Spielregeln und Praktiken in neue Formate der Distanz und des Abstands. Das gilt besonders im Superwahljahr 2021, in dem eine strategische politische Kommunikation der Mobilisierung für Parteien und Personen zwingend notwendig ist. Wir fühlen uns bei den Kulturtechniken der Demokratie in außergewöhnlicher Weise herausgefordert, oft auch überfordert. Informieren, organisieren, erinnern, kommunizieren, partizipieren, mobilisieren, debattieren – all das gilt in der Früh-Digitalisierung unseres Alltags ohnehin schon seit einigen Jahren als neues Betriebssystem unserer Gesellschaft (Borucki et al. 2020). Altanaloge Kulturtechniken der Demokratie sind durch digitale Formate ergänzt oder auch vollständig in diese überführt worden. Aber die Distanz-Formate galten nie ausschließlich. Das Virus veralltäglicht rasant diese Praktiken des Onlinen. Das ist durchaus auch positiv, denn dank der Digitalisierung können wir auch politisch weiter agieren, wenn Bewegungen und Begegnungen eingeschränkt sind oder Protestversammlungen coronabedingt verboten sind. Um so mehr benötigen wir Übersetzungshelfer und Moderatoren, die das neue Zeichensystem für die Bürgerinnen und Bürger anwendbar machen. Das Kommunikations-Repertoire ist vielfältiger. Immer weniger sind wir Mitglieder des Gemeinwesens. Immer häufiger Follower. Die neue Grammatik der Politik steckt noch in Erprobungsräumen, um die neuen Muster für alle verständlich und nachvollziehbarer zu machen (Korte 2019).

Und zeitgleich wächst die Sehnsucht nach verläßlicher Autorität, das große Ganze stellvertrend zu ordnen und idealerweise neue Orte des Gemeinwohls zu schaffen. Wer bietet Moral-Währungen als Ressourcen des Vertrauens in diesem neuen Betriebssystem an? Wo ist mein Ort, an dem ich wertgeschätzt werde, wo ich das Gefühl haben, meinen Platz zu finden? Wo bestehen Möglichkeiten, gemeinsame Einschätzungen von Problemen und Priorisierungen wahrzunehmen? Was tun Parteien im Bundestagswahljahr, um diese Fragen zu beantworten? Auch Meinungsbildung ist in der Distanz sehr schwer. Willensbildung geht oft einher mit Group-Thinking. Die Logik des Sozialen, die interpersonale Kommunikation, das Erlebnis der Begegnung formt Meinungen (Podschuweit und Geise 2015). Auch das fehlt uns im Moment, so dass wir umsomehr mit uns selbst beschäftigt sind. Orientierungsnomaden garantieren aber keine verläßliche Stimmabgabe bei Wahlen, die im Ergebnis in der Regel seit Jahrzehnten die politische Mitte in Deutschland stärkten und ausdifferenzierten. Zudem geizen die Formate von Videokonferenzen systematisch mit Resonanz. Selten ist man so allein, wie beim Vortragen ins Dunkel des Nichts.

## Kuratiertes Regieren als Politikmanagement

Prägt sich in diesen Konturen des Neuen, mit diesem veränderten Betriebssystem des Politischen, in einer digitalen Eiligkeitsgesellschaft eine Zukunft aus (Florack et al. 2021)? Kehren wir nach der Pandemie zur Nähe-Demokratie zurück? Ist das Bundestagswahljahr dafür ein Kipp-Punkt? Das kann man zum jetzigen Zeitpunkt nicht sagen. Was sich jedoch zeigt, ist, dass unser politisches System ganz offenbar mit der pandemischen Disruption zurecht kommt. Die Gewaltenteilung funktioniert. Die Zustimmung der Bevölkerung zu den Parteien der Mitte ist unverändert hoch – mit nachlassender Tendenz bei versuchter politischer Moderation von Ungeduld. Die Akzeptanz der politischen Führung ist ebenfalls ausgeprägt hoch. Das spricht ganz offenbar für eine belastbare Zukunftsfähigkeit unserer Demkratie? Die Corona-Politik hat paradigmatische Züge im Kontext einer begrenzten Ausnahmesituation. Die Zentralität der Entscheidungen zu Beginn der Pandemie, die monothematische Zuspitzung als Total-Reduktion von Komplexität, das Ausmaß des angeordneten Lockdowns für alle Lebensbereiche sowie die Einschränkungen elementarer Freiheitsrechte waren vorbildlos neu und grundlegend. Keine Krise seit 1949 hat jemals zuvor zeitgleich alle Bürger betroffen.

Zukunftsfähigkeit des politischen Systems entscheidet sich allerdings am Grad der Resilienz. Lassen wir resilienzbildende Ressourcen in der Krise zu, um das Unwahrscheinliche zu managen? Wir benötigen ganz offensichtlich die Dramaturgie von positivem Risikowissen und von Krisenprävention. Zu den Bausteinen von unterschiedlicher Qualität gehören Prozesse des Selbstlernens und der Fehlerfreundlichkeit, die Kraft der Dezentralität und Vielfalt, epistemische Ressourcen, entscheidungsfähig bleiben, zuversichtliches Erwartungsmanagement und Krisen-Lotsenschaft des politischen Personals. Diese Bausteine navigieren eine Demokratie durch viele Krisen von unterschiedlicher Qualität und Herausforderung. Die Corona-Krise informiert uns über realisierte Risiken. Ein zukünftig risikoloser Umgang mit neuen Herausforderungen resultiert daraus sicher nicht. Aber die Bewältigung von Krisen, welche die Strukturen der Resilienz klug nutzen, ist aussichtsreich unter den Bedingungen einer Ordnung der Freiheit. Die Resilienzerfahrungen bilden eine Art Antikörper in Form von Risikowissen aus und stärken das politische Immunsystem (Jage-Bowler 2020).

In Deutschland herrschten Kontaktbeschränkungen, nur in wenigen Ausnahmefällen Ausgangssperren. In den Begrifflichkeiten transportiert sich das Verständnis von sogenanntem „kuratiertem Regieren“: kein umfassendes Verbots- und Regulierungsregime, sondern eine Steuerung zwischen markteinschränkender Regulierung (durchaus mit Verboten und Marktausschlüssen/Schließungen etc.) und weichem Paternalismus, der appellativ formen soll^2^. So etablierte sich Solidarität als eine Art zivilgesellschaftlicher Selbstkontrolle, an welche die Kanzlerin in ihrer legendären Fernsehansprache besonders appelliert hatte. Konformes Verhalten wird in der Gesellschaft und durch die Politik prämiert, abweichendes Verhalten sanktioniert. Kuratiertes Regieren zielt auf eine Verhaltenssteuerung – im Fall von Corona durchaus auch auf kollektive Moralität. Denn der Rechtsstaat kann nicht mit Gewalt erzwingen, dass Gesundheit den höchsten Stellenwert im Handeln der Bürger erreicht. Gleichzeitig wächst die Erkenntnis, dass klare Regeln, die nicht nur kommuniziert werden, sondern auch von den Parlamenten erstritten sind, Freiheit schaffen. Nur diese Voraussetzung sichert die Akzeptanz der Corona-Politik. Regeln zu regeln, gehört zum Fundament des Optimismus, politisch die Krise zu meistern. Die Zuversicht, nicht die Angstmache, stabilisiert kuratiertes Regieren.

## Mobilisierungsangebote zum Vorsorgestaat

Für das Superwahljahr 2021 stellen sich naheliegende Mobilisierungsherausforderungen. Im Moment hoffen wir, im September eine geimpfte Republik wählend zu erleben. Die infizierten Blicke haben wir uns keineswegs dann bereits abgewöhnt, dennoch spielen Perspektiven eine größere Rolle als vertane Chancen. Rückblickend werden sich Wähler fragen, warum die Politik nicht ausreichend für den Pandemie-Fall vorbereitet war? Können Schuldige ausfindig gemacht werden? Zukunftsmobilisierend spielen hingegen eher die Themen eine Rolle, die strategisch Vorsorge ins Zentrum rücken und damit das Primat des Politischen. Kluge Strategen stärken alles, was zum Vorsorgestaat gehören sollte. Was soll über eine agile und kritische Infrastruktur hinaus an Daseinsvorsorge im existentiellen Bereich erhalten bzw. aufgebaut werden? Die Parteien müssen hierauf konkrete Sicherheitsbotschaften geben, die sich über traditionelle Glaubenssätze hinwegsetzen. Denn die Konturen des Neuen haben trotz mehrheitlich Status Quo orientierter älterer Wählerschichten diesmal eine realistische Chance. Denn wir haben uns seit dem ersten Lockdown nicht nur an fundamentale Transformationen gewöhnt, sondern auch erfahren, dass offenbar alles potenziell geht, was wir bislang für nicht möglich gehalten hatten. Das gilt für die Schließung von Grenzen, über Schuldenbremsen bis hin zur Präsenzkultur in Firmen. Wer insofern Veränderungen predigt, die auf inklusive Transformationen als enkeltaugliche Modernisierung unserer Gesellschaft zielen, wird bei der Bundestagswahl mehr Zuhörer haben als noch 2017. Wer sich dabei besonders um politische Verlassenheit im ländlichen Raum kümmert, wird auf Resonanz stoßen und gleichzeitig Vorsorge gegen politischen Extremismus betreiben. Krisengewinner können Möglichkeitsmacher sein mit konkreter Zuversicht. Entschlossene Krisenlotsen sind immer auch Hermeneuten der Wut, gerade in Zeiten, in denen Wutvorräte auf den Straßen und in den sozialen Medien offensiv abgebaut werden. Politiker, die übersetzen, moderieren und Zukunft erzählen, profitieren. Idealerweise geben sie der Rettung eine Richtung für demokratisch legitimierte Politik. Ihr Politikmanagement folgt dabei dem Modus des kuratierten Regierens – als Muster von Resilienz. Wer von den Parteien und Kanzlerkandidaten findet die Tonalität und den Plot für diese große Erzählung?

Die Pandemie stellt auch die Forschung vor neue Herausforderungen. Uns fehlen angesichts der Ausnahmezeiten die Vergleichsmaßstäbe. Wie wirkt sich die stillgestellte Zeit aus? Welche neuen Formate der Mobilisierung verstärken die eigene Anhängerschaft? Auch die Erkenntnissuche muß mit dem Unwahrscheinlichen rechnen. Denn Umgangsroutinen sind trügerisch. Festhalten am Bewährten ist in diesen Zeiten ein sicherer Weg zum Scheitern.

## Die Bundestagswahl als Pandemie-Solitär

Drei Besonderheiten lassen sich für die Bundestagswahl vorsichtig formulieren. Die *erste* ist: die Dominanz dieser komplexen pandemischen Lebensumstände entscheiden ganz sicher den Ausgang der Bundestagswahl. Wir wissen allerdings nicht in welcher Richtung. Denn die Auswahl der Kandidaten wird bestimmt vom Auftritt der Krisenlotsen. Niemals wäre Olaf Scholz so früh von der SPD zum Kanzlerkandidaten gekürt worden, wenn er nicht als Bundesfinanzminister eine so sichtbar dominante Rolle als Krisenmakler gespielt hätte. Ohne das Virus wäre vermutlich auch Armin Laschet nicht Parteivorsitzender geworden. Das Virus prägt die Themen und fächert den Parteienwettbewerb auf. Ohne das Virus hätte vermutlich auch die AfD einen Hauch von bürgerlichem Rückhalt bei einigen Wählern behalten.

Damit Krisenmanagement funktioniert, sind Entscheidungsträger nicht nur auf ihre Entscheidungskompetenz angewiesen, sondern auch auf Bürgerinnen und Bürger. Sie brauchen Vertrauen in die Funktionsträger und deren Risiko- und Lösungskompetenz. Bürger müssen sich sowohl an die Einschränkungen als auch an die Appelle halten. Erstaunlich war insofern, dass eine diffuse, jahrelang anhaltende Politikverdrossenheit unter den Bedingungen der Corona-Politik wie weggefegt wirkte. Nie zuvor war die Staatsgläubigkeit so hoch und das akzeptierte Verständnis für die massiven Einschränkungen von Freiheiten so breit. Massive Einschränkungen bürgerlicher Freiheiten (Gewerbefreiheit, Reisefreiheit, Versammlungsfreiheit) wurden widerspruchslos mehrheitlich hingenommen. Das Coronavirus hat dem Staat nicht nur mehr Regelungsmacht im Katastrophenfall gegeben, sondern katapultierte ihn auch zum rhetorisch-emotionalen Krisengewinner. Historisch zeigt sich: Der Grad an Staatszentriertheit und Staatsvertrauen nimmt in Deutschland zu, wenn Krisenszenarien die öffentliche Meinung dominieren.

Davon profitieren auch viele Spitzenpolitiker. Krisen adeln über Nacht die Demokratie und die Spitzenpolitiker. Bürger erwarten dann die entschlossene Umsetzung des Primats der Politik, möglichst als heroische Chefsache des Krisenmanagers. Die Bürger sehnen sich in solchen Konstellationen nach einem starken Staat und erwarten dann die entschlossene Umsetzung des Primats der Politik. Die Sehnsucht nach Etatismus^3^ breitet sich besonders dann aus, wenn Verlustängste dominieren. Sicher haben auch mittlerweile zwei Signalereignisse, diese über Monate stabil hohen Zustimmungswerte der Bürger zur Corona-Politik eingetrübt. Dazu zählen das desaströse Erwartungsmanagement beim Impfstart sowie der plötzliche Austausch von Zahlen – aus dem monatelang gepredigten Inzidenzwert von 50 wurde plötzlich eine 35. So bröckelten Zustimmungswerte zum Corona-Management der Regierungen. Dennoch kann man zum jetzigen Zeitpunkt davon ausgehen, dass einige der Corona-bedingten Einschränkungen bis September 2021 aufgehoben sein werden, was den Protest einmal mehr minimiert.

Eine *zweite* Besonderheit – neben der Pandemie – gilt der historischen Konstellation: Niemals zuvor fanden Bundestagswahlen ohne Titelverteidiger statt – sieht man von 1949 einmal ab. Bundeskanzlerin Merkel verzichtete auf eine erneute Kandidatur und gab den Parteivorsitz ab. Sie hat jetzt die realistische Chance, würdevoll bedeutungslos zu werden. Sowas gehört im politischen Geschäft zur allergrößten Herausforderung. Der Merkel-Bonus, als ein Plus für die Corona-Titanin der Union, katapultierte die Union ab April 2020 zu neuen Höchstwerten bei der Sonntagsumfrage. Je deutlicher wird, dass Merkel in 2021 auf keinem Wahlzettel stehen wird, steigen die Chancen der Mitbewerber, vor allem der Grünen und der SPD. Alle drei Parteien stehen nebeneinander auf der Startlinie, um für Programm und Personen zu werben. Kommende TV-Trielle dokumentieren im Format die Innovation 2021, einer Bundestagswahl ohne Verteidigerin. Die Koalitionsvarianten lauten deshalb aus Sicht des Frühjahrs 2021: Schwarz-Grün als mögliche Fortsetzung der Großen Koalition (als Bündnis der beiden größten Bundestagsfraktionen); Schwarz-Grün-Gelb als Jamaika revival; Grün-Rot-Gelb als grüne Ampel (in dieser Formation ein Solitär). Eine Corona-Prämie wird am Wahltag nur zu verteilen sein, wenn die Pandemie überwunden scheint. Falls nicht, könnte eine All-over-Koalition (ohne AfD) als Not-Regierung erforderlich werden – ein sehr unwahrscheinliches Szenario. Das politische Ende von Merkel markiert auch eine kulturelle Zäsur für sehr viele Wählerinnen und Wähler. Mit ihr geht ein verläßlicher Vertrauensanker für den Stoff des Politischen verloren. Wem bringen Wähler diesen Vertrauensvorschuß zukünftig entgegen? Neue Koalitionen wiederum bilden nicht nur Stimmungen ab, sondern prägen auch neue. Sie setzen in der Regel gemeinsame neue Ideen und eine neue gesellschaftliche Dynamik voraus. Das gilt sowohl konzeptionell, personell als auch intellektuell. Wichtig bleibt auch, dass sie sich anbahnen mit Spürgefühl und nicht nur eine Folge von rechnerischen Mehrheiten sind.

Eine *dritte* Besonderheit liegt ebenso im historischen Vergleich der Wahlen. Am Ende der Adenauerzeit existierte ebenso wie nach 16 Jahren Kohl ein starker Wunsch nach Veränderung, nach Wenden, nach Neuanfang. 2021 ist eine Wechselstimmung bislang nicht meßbar. 16 Jahre Merkel mit drei Großen Koalitionen führen keinswegs zum überwölbenden und eindeutigen Wunsch nach neuen Formaten der Macht, nach einem neuen Führungs- und Kooperationsstil, nach neuen Möglichkeiten des guten Regierens (Korte et al. 2018). Noch herrscht Wirklichkeitsgehorsam. Noch prägt die Erinnerung an staub-trockene Krisenpolitik als letzte Variante einer sogenannten Alternativlosigkeit. Die Option, wie 1998, zwei Oppositionsparteien in die Regierungsverantwortung zu katapulieren, erscheint abwegig. Zwar existiert meßbar auch ein Überdruß an der Alltäglichkeit des wegmoderierenden Pragmatismus, dem unterargumentierenden Regieren und der stets situativen postheroischen Empörungsverweigerung. Doch die Sehnsucht nach der großen empathischen Erzählung, den Lotsen der schonenden Transformation ist noch nicht so ausgeprägt, dass ein „Weiter-So“ mit anderem Personal vollkommen ausgeschlossen wäre. Als besonders Merkel-enkeltauglich erweist sich der Kandidat der SPD. Scholz steht, ebenso wie die Kanzlerin, für die gesellschaftspolitische progressive Mitte. Er hat in Hamburg bewiesen, wie moderne Urbanität sozialverträglich mehrheitsfähig bleibt. Als Typus prägt er auch das Ruhe-Regiment, mit vornehmer Unangreifbarkeit, Solidität und Risiko-Unlust. Wer sich für die Fortsetzung der Merkel-Politik stark macht, findet mit Scholz einen sehr mächtigen Aspiranten. Wenn Wähler weiterhin auf das Bekannte und weniger auf das Unbekannte setzen, könnte Scholz den Vizekanzler-Bonus voll einbringen.

Die Grünen leben vom Zulauf aus mehreren Richtungen. Sie sind multikoalitionsfähig – sichtbar in Regierungsverantwortung und in der Opposition zugleich. Sie kratzen an der seit Corona hegemonialen Dominanz der Union. Sie verkörpern das Kompetenzzentrum für Umwelt- und Klimapolitik, einem Thema, welches bürgerliche Wähler sehr beschäftigt. Das Virus hat gezeigt, dass nicht wir, sondern die Natur uns im Griff hat. Ein schonender Umgang mit Ressourcen in der stillgestellten Zeit hat bürgerliche Wähler zusätzlich mit grünen Ideen versöhnt. Von der Corona-Prämie profitieren die Grünen, weil sie auch mit ihrer professionellen Doppelspitze im Bund einen gewachsenen Bedarf nach normativer Orientierung befriedigen. Sie setzen mit ihrer eigenen Moral-Währung voll auf die schonende und gemeinsame Transformation der Gesellschaft. Der Kommunikations- und Führungsstil begeistert bürgerliche Kreise, die sich mit Realitäts-Demut geißeln. Hier hat nicht die neo-dirigistische Entschiedenheitsprosa Aussicht auf Gehör, sondern eher Macht-Poesie als Moderation von Ambivalenzen. Zwanzig Prozent der Wählerschaft sind damit abholbar. Das Grüne Verständnis von hybriden gesellschaftlichen Bündnissen wird vermutlich auch eine Festlegung auf nur einen Kanzlerkandidaten verhindern. Unsere Neugierde wird bis zuletzt bleiben, wie sich das Tandem bei einer möglichen Koalitionsverhandlung am Ende einigt. Wähler der Grünen brauchen keine Festlegung vorab. Sie vertrauen der klugen Machtteilung, wenn es nicht nur um Ehrentitel geht, sondern um konkrete Ressorts. Letztlich werden die Grünen immer nach der Kanzlerschaft greifen, wenn es sich mit welchen Koalitionspartnern auch immer (außer der AfD) rechnerisch lohnt. Eine allzu selbstsicher agierende Union könnte am Ende, trotz größter Fraktion im Bundestag, in der Opposition landen.

Der liberale Wählerblock steht auch für die FDP zur Verfügung, gleichwohl auf niedrigerem Niveau als bei den Grünen. Moderne Autonomie, moralischer Ernst, bürgerliche Solidität und gemeinwohlorientierter Kaufmannsgeist finden sich auch bei der FDP. Grüne und gelbe Liberale haben viele Schnittmengen. Sie spielten nur früher an ganz unterschiedlichen Stellen auf den Schulhöfen – nie zusammen. Sie trennen Milieus und Auftritte, weniger die Fragen der Liberalität. Aber im Dilemma-Management zwischen Freiheit und Gesundheit, setzen sich die FDP, anders als die Grünen, deutlicher für die Freiheit in der Abwägung ein. Grundsätzlich agiert die FDP in der Corona-Politik aus der Defensive. Denn staatsbeseelte Wähler, die darauf setzen, dass der Staat sie persönlich und finanziell schützt und rettet, kann eine Partei, die mehr auf individuelle Freiheit setzt, nur schwer einsammeln. Die Linke wird dann profitieren, wenn die Post-Corona Zeit zu größeren sozialen Verwerfungen führen könnte, was allerdings Berlin mit hohem finanziellen Aufwand versucht, zu verhindern. Grundsätzlich hat politisch linkes Denken in der Pandemie Zulauf: Staat statt Markt klingt dabei wie eine resilienzermöglichende Perspektive. Es zeigt, wie sich weltweit das ökonomische Denken auf der links-rechts Achse nach links verschoben hat.

Das Superwahljahr folgt einer ausseralltäglichen Logik. Es bleibt eigenartig einzigartig. Die Grundstimmung changiert zwischen einem Enthusiasmus des Positiven und der Wehmut des Vorsichtigen: sorgenvoll zufrieden oder zufrieden im Unbehagen? Das stellt Wahl‑, Parteien, Regierungsforschung vor besondere Herausforderungen. Die gewählten Parteien folgen im Setting des Bundestages vermutlich erneut einem polarisiertem Pluralismus. Die Radikalisierung im Parteienspektrum bleibt der AfD vorbehalten, die lösungsorientiert zur Corona-Politik wenig beizutragen hat. Die politische Mitte wird sich weiterhin ausdifferenzieren, aber nicht kleiner, sondern eher größer werden. Ob der Bundespräsident erneut der zentrale Kanzlermacher einer neuen Regierungsformation sein wird, bleibt spekulativ (Korte 2019). Mit seiner Reservemacht steht er mehr denn jemals zuvor als Möglichkeitsmacher bereit, sollte sich eine blockierte Regierungsbildung abzeichnen. Mit Corona-Kreativität und ohne die Corona-Titanin wird er dann Auswege finden müssen.

Sorgenvolle Zufriedenheit? Was folgt aus dieser Stimmung des Zufriedenseins („wir haben es geschafft“) im Unbehagen (vom falschen Erwartungsmanagement der Bundesregierung bis zum Mißmanagement bei den Lockerungen)? In solchen Zeiten kommt der Rolle der Demoskopie eine zusätzliche Bedeutung als strategisches Potenzial der Wahlkampfführung zu. Denn sie formiert nicht nur Momentaufnahmen – auch des sozial Erwünschten – sondern sie fixiert auch Haltepunkte in einer extremen Zeit der Ungewißheit mit einem unsichtbaren Feind.

## Footnotes

1 Teile des nachfolgenden Essays erscheinen in der Zeitschrift „Politische Studien“ Heft Mai/Juni 2021.

2 Zu den dahinterliegenden Mechanismen des Entscheidens sind bald im Kontext eines Forschungsprojekts Ergebnisse nachzulesen: Korte et al: Politische Heuristiken, Berlin (2021).

3 Man könnte den Begriff etwas bösartig als „vorauseilende Unterwürfigkeit“ übersetzen.
